# New Interleukin-23 Antagonists’ Use in Crohn’s Disease

**DOI:** 10.3390/ph18040447

**Published:** 2025-03-22

**Authors:** Laura Biskup, Jan Semeradt, Jagoda Rogowska, Wiktoria Chort, Łukasz Durko, Ewa Małecka-Wojciesko

**Affiliations:** Department of Digestive Tract Diseases, Medical University of Lodz, 90-419 Lodz, Poland

**Keywords:** Crohn’s disease, IL-23 inhibitors, risankizumab, guselkumab, mirikizumab, inflammatory bowel disease

## Abstract

Crohn’s disease (CD) is a chronic inflammatory condition of the digestive tract, driven by an imbalance in immune system regulation, where proinflammatory interleukin-23 (IL-23) plays an essential role. Selective new IL-23 inhibitors, including risankizumab, guselkumab, and mirikizumab, block the IL-23p19 subunit to inhibit the Il-23 action and alleviate inflammation in CD. This review explores the effectiveness, safety, and therapeutic potential of anti-IL-23 treatment in CD management. Risankizumab, guselkumab, and mirikizumab demonstrated considerable effectiveness in inducing clinical remission and promoting endoscopic healing in patients with moderately to severely active CD, including those refractory to anti-TNF therapies. Risankizumab showed favorable results in pivotal trials like ADVANCE, MOTIVATE, and FORTIFY, achieving remission rates of up to 45% and sustained inflammatory biomarkers normalization. Guselkumab and mirikizumab similarly demonstrated substantial efficacy in the induction and maintenance phases, with promising long-term results. The safety profiles of IL-23 inhibitors were favorable, with low rates of serious adverse events, including infections and malignancies. Selective new IL-23 inhibitors represent a targeted and effective therapeutic class for moderately to severely active CD, offering high clinical and endoscopic remission rates, and favorable safety outcomes. Continued research, particularly on long-term efficacy and the selection of patients based on inflammatory biomarkers, will help optimize their role in personalized treatment strategies for refractory CD.

## 1. Introduction

CD is a long-lasting, relapsing inflammatory bowel disease (IBD) induced by the complex interplay of hereditary, environmental, and immunological influences. It primarily affects the gastrointestinal (GI) tract, with inflammation that can occur anywhere from the oral cavity to the anus, but most commonly impacts the terminal ileum and colon [1]. The disease is marked by remission and relapse periods, with varying symptoms depending on the site and severity of inflammation. Symptoms often include abdominal pain, diarrhea, fatigue, and weight loss, and over time, the CD can lead to issues like strictures, abnormal passages, and local infections [2,3]. CD pathophysiology is driven by an overactive immune response to the different external and internal factors, including gut microbiota in genetically susceptible individuals [2]. Dysregulated T-cells, particularly CD4^+^ T-cells, play a key role in sustaining inflammation, leading to epithelial damage in the intestine. These cells are predominantly skewed toward pro-inflammatory subsets, such as Th1 and Th17, which release cytokines like IFN-γ and IL-17. IFN-γ disrupts tight junctions in epithelial cells, increasing intestinal permeability and enabling microbial antigens to trigger further immune activation. IL-17 promotes neutrophil recruitment and the release of matrix metalloproteinases (MMPs), leading to tissue destruction and impaired repair. At the same time, regulatory T-cells fail to suppress this overactive response, allowing inflammation to persist and exacerbate damage [4]. Additionally, gut microbiota species, such as *Mycobacterium avium* subspecies *paratuberculosis* (MAP), have been shown to contribute to chronic inflammation [3]. MAP has been frequently detected in the blood, feces, and intestinal specimens of Crohn’s disease patients at significantly higher rates compared to controls. It survives within macrophages by resisting degradation in phagolysosomes, leading to the secretion of cytokines driving inflammation, including tumor necrosis factor- α (TNF-α), which perpetuates inflammation. MAP also disrupts autophagy, a critical cellular process for clearing intracellular pathogens and maintaining epithelial integrity. This impairment can lead to increased bacterial persistence and dysregulated immune responses [5]. AIEC (*adherent-invasive Escherichia coli*) is frequently found in the ileal mucosa of Crohn’s disease patients, showing the ability of adherence and invasion of the epithelial cells, aided by overexpressed CEACAM6 receptors in inflamed tissues. Once inside, AIEC replicates within vacuoles, evades immune defenses, and disrupts tight junctions, weakening the intestinal barrier. This allows luminal antigens to penetrate the mucosa, driving immune activation. Additionally, AIEC induces excessive production of IL-8, promoting neutrophil infiltration and chronic inflammation, which exacerbates tissue damage [6].

Genetic predispositions, including polymorphisms in autophagy-related genes like *NOD2* and *ATG16L1*, have also been identified, further complicating the immune response and contributing to the chronicity of CD [7].

With its unpredictable course, CD poses significant challenges in disease management and places a considerable burden on patients’ quality of life and healthcare systems worldwide [8].

Traditional treatment strategies for CD have largely focused on symptom control and inflammation reduction, using glucocorticoids (GCSs), immunosuppressants, and tumor necrosis factor (TNF) inhibitors [9]. While these therapies have shown efficacy in managing acute inflammation and inducing remission, they are often limited by significant side effects, variable long-term efficacy, and the risk of treatment resistance [10]. GCSs, for instance, are widely used for short-term symptom control but are associated with numerous adverse effects [11]. Similarly, immunosuppressants such as azathioprine and methotrexate, although beneficial, have narrow therapeutic windows and can lead to increased susceptibility to different infections, including *Herpes zoster*, CMV, JC virus (PML), hepatitis virus B and C, and exacerbation of *P. jirovecii*, and *Nocardia* [12]. Patients are also prone to experience fatigue, abdominal pain, increased stomach irritation, development of mouth sores and ulcers, loss of appetite, and a higher risk of developing certain tumors, mainly skin cancer [13].

TNF inhibitors, biologic drugs targeting TNF-α, have revolutionized Crohn’s disease treatment by the significant reduction in inflammation and mucosal healing promotion, particularly in moderate to severe cases. Agents like infliximab and adalimumab marked a shift from broad immunosuppression to targeted therapy. However, they are not the only biologics; newer options like IL-12/23 and IL-23 inhibitors have since emerged. On the other hand, challenges such as loss of response, primary non-response, and side effects highlight the need for more precise and durable treatment strategies [14]. It has been observed that more than 30% of patients do not respond to TNF-α antagonists initially, and up to 50% may lose their response over extended follow-up [15]. In addition, TNF-α antagonists have been linked to a higher risk of opportunistic infections and lymphomas in patients with IBD, particularly when used in conjunction with immunosuppressive therapies.

Given these limitations, there is an increasing need for innovative and targeted therapies to address the unmet needs in Crohn’s disease management [16]. Current therapies often fail to fully target inflammation or promote mucosal healing, leaving patients vulnerable to complications like strictures and fistulas. Additionally, managing extraintestinal manifestations, improving quality of life, and reducing the reliance on surgery remain significant challenges. Innovative therapies, such as IL-23 inhibitors, hold promise in addressing these gaps by offering more targeted and effective options for disease control [16]. This article aims to explore emerging treatment strategies in CD care, focusing specifically on IL-23 inhibitors, including risankizumab, guselkumab, and mirikizumab. These targeted biologics represent a significant advancement in addressing the inflammatory pathways central to Crohn’s disease pathogenesis, offering new hope for improved disease control and patient outcomes [17]. Focusing on recent advancements, such as newer biologics, small molecules, and precision medicine approaches, we will discuss the potential to improve long-term outcomes and provide a more personalized approach to treatment [18].

### Methodology

For this review, data were gathered from multiple electronic databases, including PubMed, Embase, Cochrane Library, and Web of Science, to ensure a comprehensive evaluation of the available literature. The search terms included “Crohn’s disease”, “IL-23 inhibitors”, “risankizumab”, “guselkumab”, “mirikizumab”, and “inflammatory bowel disease”. Articles published between 2018 and 2024 were selected to focus on recent advancements in treatment.

The review prioritized human studies, particularly clinical trials, systematic reviews, and meta-analyses that assessed the efficacy and safety of IL-23 inhibitors in managing Crohn’s disease. The selection process followed predefined inclusion and exclusion criteria, ensuring the relevance and quality of the studies analyzed. To minimize bias, the risk of bias assessment was conducted using validated tools, such as the Cochrane Risk of Bias Tool for clinical trials and the Newcastle–Ottawa Scale for observational studies.

Extracted data focused on therapeutic interventions, clinical effectiveness, safety profiles, and the impact of these treatments on disease activity and patient quality of life. A structured approach was applied to data extraction and synthesis to ensure transparency and reproducibility in the review process.

## 2. The Involvement of IL-23 in the Development of Crohn’s Disease

IL-23 is a heterodimeric cytokine which belongs to the IL-12 cytokine family, along with IL-12, IL-27, and IL-35. IL-12 and IL-23 take part in proinflammatory reactions, with IL-23 playing a pivotal role in tissue damage. The disruption of IL-23 and its receptor pathways is seen in a variety of inflammatory disorders, such as IBD, psoriasis, and cancer [19].

IL-23 is secreted by antigen-presenting cells (APCs), mainly macrophages and dendritic cells (DCs). It consists of an IL-12p40 component, which is shared with IL-12, and a specific IL-23p19 component. IL-23 is a key activator of pathogenic Th17 cells in many chronic autoimmune conditions [20]. These Th17 cells extensively infiltrate the affected intestine of patients with CD, where they secrete inflammation-promoting cytokines such as IL-17, IL-22, and GM-CSF (granulocyte-macrophage colony-stimulating factor). IL-23 itself promotes the release of several proinflammatory cytokines—TNF, IL-1β, IL-6, IL-8, and IL-10 [21].

IL-23 engages with two receptors: the specific IL-23 receptor (IL-23R) and IL-12Rβ1 [19]. The IL-23R is expressed on lymphoid cells, CD4^+^ T helper 17 (Th17), which are believed to be crucial in CD pathogenesis [19], and CD8^+^ T cells [21]. Increased expression of IL-23R has been shown in lamina propria CD4^+^ mucosal cells in the small and large intestine of CD patients with quantitative polymerase chain reaction (PCR) amplification [22]. The binding of IL-23 to its receptor triggers a conformational shift that activates two cytoplasmic tyrosine kinases: Janus kinase 2 and tyrosine kinase 2 (TYK2). This initiates multiple pathways that play a central role in inflammation, including P38 MAPK, PI3K-Akt, or the NFкB pathway. This process leads to the secretion of cytokines connected with CD: IL-17A, IL-17F, or IL-22 [23].

After exposure to specific stimuli (such as microbial signals, cytokines, and co-stimulatory T-cell ligands), DCs and macrophages increase the production of IL-23. Macrophages CD14^+^ CD33^+^ in the lamina propria of the intestine are particularly prone to producing IL-23 upon exposure to bacterial strains, such as *Escherichia coli* and *Enterococcus faecalis*. Therefore, intestinal microbiota have been shown to play a role in driving chronic inflammation, increasing Il-23 synthesis. The number of *Escherichia coli* and *Enterococcus faecalis* strains was higher in CD patients than in healthy subjects [19].

Data collected from in vitro studies, animal models, and human genetic association research confirm the involvement of IL-23 in the pathogenesis of CD and other immune-driven disorders [21]. A genome-wide association study (GWAS) examined the polymorphism in the gene that encodes IL-23R., which was conducted using 308,332 autosomal single nucleotide polymorphisms (SNPs). The GWAS focused on 547 cases of ileal CD, the most common subtype, and 548 controls. Three SNPs were found to be significantly associated with CD in single-marker allelic tests, including rs11209026 in the IL-23R gene located on chromosome 1p31, encoding a component of the IL-23 receptor [24].

Intestinal inflammation was observed in mice with transgenic overexpression of IL-23p19. This was accomplished by injecting a p19 transgene into mouse egg cells, leading to increased IL-23p19 production in the transgenic mice [25]. Mouse intestine tissues were fixed in 10% phosphate-buffered formalin, processed into 5 μm segments, and stained with hematoxylin and eosin. In the intestines, mild to moderate, multifocal inflammatory infiltrates were mainly found in the epithelium, lamina propria, and submucosa and were frequently accompanied by epithelial hyperplasia. These infiltrates comprised mainly lymphocytes and macrophages [25].

Another study has shown that the blockade of IL-23p19 in an experimental model of colitis reduced inflammation [21]. *H. hepaticus*-infected mice presented significantly increased expression of IL-23p19 mRNA (measured by real-time quantitative PCR) in the cecum and colon, compared to uninfected controls. In these mice, the amount of inflammation-promoting cytokines (TNF-α, IFN-γ, IL-6, MCP-1, IL-1β, KC, IL-17) was significantly elevated. Colon homogenates from *H. hepaticus*-infected mice that received anti-IL-23p19 were characterized by comparably low levels of proinflammatory cytokines (TNF-α, IFN-γ, IL-6, MCP-1, IL-1β, KC, IL-17) to those observed in the uninfected controls [26].

In addition, the variant of IL-23R that significantly reduces the risk of development of IBD has been detected with a GWAS. The glutamine allele (p.Arg381Gln) of IL-23R is a rare variant, protecting against CD onset in both non-Jewish populations (odds ratio (OR) = 0.26; 95% confidence interval (CI): 0.15–0.43) and Jewish populations (OR, 0.45; 95% CI: 0.27–0.73) [24]. The IL-23R^R381Q^ protective variant causes a dysfunction in IL-23R, which reduces Th17-mediated cell responses upon exposure to IL-23, therefore diminishing the key mediators in autoimmunity [21].

A newly published analysis has demonstrated that patients resistant to anti-TNF-α therapy present increased expression of IL-23R in mucosal CD4^+^ cells, expressing TNFR2, one of the two main receptors for TNF-α. These cells can evade anti-TNF-induced apoptosis by activating IL-23R. In consequence, high amounts of Th1 and Th17 are produced, which is commonly seen in CD. The TNFR2^+^IL-23R^+^ T complex accumulates in the mucosa of patients refractory to TNF-α antagonists, contributing to chronic inflammation by increasing the synthesis of proinflammatory cytokines such as IL-17, and IL-6. This implies that the presented patients’ group could especially benefit from therapies targeting IL-23 [19].

Overall, therapies targeting IL-23, specifically the p19 subunit, have several potential advantages over TNF-α antagonists, which have been broadly used in CD. IL-23 inhibitors selectively block the activity of IL-23, which can effectively reduce inflammation while minimizing unintended immune suppression. Clinical trials have shown that they can effectively induce and maintain symptom relief in CD patients, even in those who are unresponsive to anti-TNF agents. In addition to symptom control, they have been effective in promoting mucosal healing, which is crucial in CD treatment and can reduce the need for surgical interventions and hospitalizations. IL-23p19 inhibitors are linked to minimal occurrence of neutralizing antibody development [20] contrary to TNF-α antagonists, which reduces the likelihood of treatment failure. Compared to traditional management, IL-23p19 inhibitors have a lower risk of systemic side effects and serious infections. Forty-nine randomized placebo-controlled studies comprising 14,590 participants showed that patients receiving TNF-α antagonists experienced a measurable rise in the probability of any infection (OR, 1.19; 95% CI, 1.10–1.29) and a significant rise in the probability of opportunistic infections (OR, 1.90; 95% CI, 1.21–3.01) [27]. Studies revealed that relative to patients who had not been exposed to anti-TNF-α, those receiving anti-TNF-α monotherapy had a pooled Incidence Rate Ratio (IRR) of lymphoma of 1.52 per 1000 patient-years (95% CI: 1.06–2.19; *p* = 0.023) [28]. The accessible evidence does not suggest an increased probability of severe infections or cancers with anti-IL-23p19 treatments [20]. Since the clear dose–response relationship for the IL-23p19 inhibitors is not observed, therapeutic drug monitoring (TDM) is not likely to be required [20]. On the contrary, TDM is often recommended when using TNF-α antagonists in treating CD.

During the last few decades, several anti-IL-23 therapies have been elaborated and improved. The first generation of anti-IL-23 treatment involves a therapy targeting the shared IL-12p40 component, inhibiting IL-12 and IL-23 pathways.

Ustekinumab is a monoclonal antibody that binds to the p40 component common to interleukins IL-12 and IL-23, inhibiting their inflammatory pathways. It was the initial biologic therapy in this category authorized for managing moderate to severe CD, providing a new option for patients who fail to respond to conventional treatments or tumor necrosis factor inhibitors. Clinical studies have demonstrated its effectiveness in promoting and sustaining remission in these patients [29].

A key benefit of ustekinumab is its positive safety record. It is generally well-tolerated, with common adverse events including nasopharyngitis, headache, and fatigue. Unlike some other biologic therapies, ustekinumab is associated with a lower risk of serious infections and malignancies. Another benefit is its convenient dosing schedule, which involves an initial intravenous infusion followed by subcutaneous injections every 8 to 12 weeks [30].

However, ustekinumab has some limitations. Its onset of action is slower compared to other biologics, with clinical response potentially taking several weeks. Additionally, the high cost of treatment can be a barrier to access for some groups of patients. While short-term safety data are reassuring, long-term safety and efficacy continue to be monitored, as rare adverse events could emerge with extended use [31].

Despite these challenges, ustekinumab remains a significant advancement in the treatment of Crohn’s disease, offering a targeted approach with proven efficacy and a favorable safety profile. Further research and long-term data will help refine its role in clinical practice [29].

The second generation of anti-IL-23 treatments addresses selectively the IL-23p19 subunit, focusing specifically on the IL-23 pathway. This strategy has demonstrated promising results in treating CD patients [19]. In this article, we introduce the current and possible use of the following IL-23p19 inhibitors in the treatment of CD: risankizumab, guselkumab, and mirikizumab.

**Figure 1:**
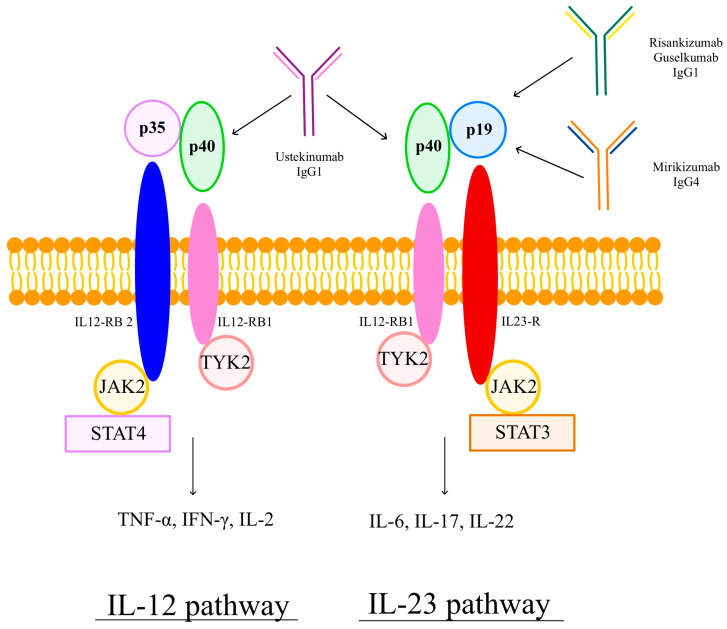
Mechanism of action of IL-23 antagonists: ustekinumab, risankizumab, guselkumab, and mirikizumab [32,33,34,35].

The mechanism of action of these IL-23 antagonists is illustrated in Figure 1.

Ustekinumab is an immunoglobulin G1 (IgG1) antibody binding to the shared p40 component of IL-12 and IL-23 cytokines, effectively inhibiting signaling in both pathways. In contrast, risankizumab (IgG1), guselkumab (IgG1), and mirikizumab (IgG4) selectively target the p19 component, unique to the IL-23 pathway. This specific mechanism blocks IL-23-mediated proinflammatory signaling while leaving the IL-12 pathway unaffected.

## 3. Pharmacological Treatment with Interleukin-23 Antagonists

### 3.1. Risankizumab

#### 3.1.1. General Information

Risankizumab is a humanized IgG1 monoclonal antibody that precisely targets the p19 component of IL-23, effectively blocking its binding to the IL-23R. This highly specific approach allows risankizumab to reduce inflammation while minimizing broad immune suppression [36]. The drug has received FDA’s approval in 2022 for managing moderately to severely active CD. It is also the first IL-23-specific inhibitor approved to treat both Crohn’s disease and ulcerative colitis [37,38]. This medication has also been effectively utilized in the treatment of plaque psoriasis and psoriatic arthritis for several years [39].

#### 3.1.2. Efficacy in Induction Therapy

The efficacy of risankizumab as an induction therapy has been studied in two phase 3 clinical trials. Both trials were similar in design; however, they differed slightly in the eligibility criteria. In the ADVANCE trial, the population included patients who either could not tolerate or failed to respond adequately to prior conventional treatments (such as corticosteroids or immunosuppressants) and/or biologic therapies. In contrast, the MOTIVATE trial focused exclusively on patients who had experienced treatment failure specifically with biologic therapies. In both studies, patients were required to have moderately to severely active disease, measured by a Crohn’s Disease Activity Index score (CDAI) between 220 and 450 at baseline. Clinical symptoms included an average daily stool frequency (SF) of four or more, or a daily abdominal pain score (AP) of at least two. To confirm active disease, endoscopic detection of mucosal inflammation was required, measured by the Simple Endoscopic Score for Crohn’s Disease (SES-CD). To be included in the studies, patients needed to have an SES-CD score of 6 or higher, or at least 4 in cases of isolated ileal disease [40].

During the ADVANCE and MOTIVATE trials, patients were randomly allocated to receive intravenous risankizumab at dosages of either 600 mg or 1200 mg, or placebo. The treatments were administered as a single dose at weeks 0, 4, and 8. Both studies demonstrated that risankizumab could achieve a rapid clinical response by week 4. This was specified as a ≥30% decrease in average daily SF, a ≥30% decrease in average daily AP, or a CDAI reduction of ≥100 points from baseline. By week 12, significant clinical remission was observed, characterized by average daily SF ≤ 2.8, AP ≤ 1, and a CDAI score < 150 points. Additionally, mucosal improvement was confirmed in endoscopy, with endoscopic remission described as an SES-CD score of ≤4 and at least a 2-point reduction from baseline. The results were consistent regardless of patients’ prior exposure to biologic therapies, suggesting that an inadequate response to other biologics does not predict a lack of efficacy with risankizumab [40].

In the ADVANCE trial, between 42% (adjusted difference (AD) 17% [95% CI 8–25]; *p* < 0.0001) and 45% of patients (AD 21% [95% CI 12–29], *p* < 0.0001), depending on the dose, achieved clinical remission based on the CDAI at week 12. Similarly, between 41% (AD 19% [95% CI 11–27]; *p* < 0.0001) and 43% of patients (AD 22% [95% CI 14–30]; *p* < 0.0001) reached remission based on SF and AP. Endoscopic response rates were also significant, with the 600 mg dose achieving a 40% (AD 28% [95% CI 21–35]; *p* < 0.0001) response rate, compared to 12% in the placebo group. Treatment with risankizumab (600 mg or 1200 mg) led to significant reductions in C-reactive protein (CRP), with 42.5% (*p* < 0.001) of patients achieving CRP levels ≤ 5 mg/L, and reductions in fecal calprotectin (FCP), where 30.1% (*p* < 0.001) of patients reached FCP levels ≤ 250 mg/kg by week 4. These improvements were sustained through week 12 [40].

In the MOTIVATE trial, similar results were observed. Between 40% (AD 21% [95% CI 12–29]; *p* < 0.0001) and 42% (22% [95% CI 13–31]; *p* < 0.0001) of patients achieved CDAI remission, while between 35% (AD 15% [95% CI 6–24]; *p* = 0.0007) and 40% (20% [95% CI 12–29]; *p* < 0.0001) reached remission based on SF and AP criteria. Endoscopic response rates for the 600 mg dose were 29% (AD 18% [95% CI 10–25]; *p* < 0.0001), compared to 11% in the placebo group. Significant reductions in CRP levels were also observed, with 34.4% (*p* < 0.001) of patients achieving CRP ≤ 5 mg/L between weeks 4 and 12. However, reductions in fecal calprotectin were significant only in the 600 mg group, where 20.1% (*p* = 0.014) of patients achieved FCP ≤ 250 mg/kg by week 12 [40].

The benefits of risankizumab therapy were also reflected in a reduced number of hospitalizations during the induction phase. The incidence of CD-related hospitalizations over 12 weeks was significantly lower in patients receiving risankizumab compared to placebo in both the ADVANCE and MOTIVATE trials. In the pooled analysis, hospitalization rates were 3.2% in the risankizumab 600 mg group and 1.9% in the risankizumab 1200 mg group, compared to 11.6% in the placebo group (*p* < 0.001). Among hospitalized patients, the proportion undergoing surgery varied, with 35.3% in the risankizumab 600 mg group, 20.0% in the risankizumab 1200 mg group, and 21.4% in the placebo group [41].

In both the ADVANCE and MOTIVATE trials, risankizumab demonstrated strong efficacy, including significant clinical and endoscopic outcomes. Notably, it proved effective in achieving mucosal healing, a crucial indicator linked to better long-term outcomes, including lower relapse rates and reduced risk of complications. The treatment was well-tolerated across patient populations, with no serious safety risks identified. However, some limitations should be noted. The requirement to maintain stable corticosteroid doses during the induction period prevented an evaluation of early steroid tapering with risankizumab. Additionally, the small number of adolescents in the trials limits the applicability of findings to younger patients.

Similar results regarding the efficacy of risankizumab were found in a GETAID retrospective cohort study, which analyzed data from 100 patients with highly refractory luminal Crohn’s disease. All patients included in the study had been previously treated with anti-TNF medications, 94 with vedolizumab, and 98 with ustekinumab. At baseline, 44 patients were on simultaneous steroid therapy, while by week 12, 29 patients had successfully discontinued steroids. Following a 12-week induction phase with 600 mg of intravenous risankizumab, 78.5% of the patients achieved a clinical response, characterized by a reduction of at least 3 points in the Harvey–Bradshaw Index (HB) or HB < 5. Clinical remission (HB < 5) was achieved in 58% of the patients, with 45.8% reaching steroid-free clinical remission. Notably, among those with prior ustekinumab treatment failure, steroid-free clinical remission rates were 40% for primary non-responders and 57% for those who had previously lost response to ustekinumab. According to patient-reported outcomes (abdominal pain and daily stool frequency), 50% of patients achieved clinical remission (defined as AP ≤ 1 and SF ≤ 3), while 39.5% of patients attained steroid-free clinical remission. Additionally, 25 patients underwent endoscopic and/or radiological evaluations at week 12, revealing that four patients achieved remission (defined as the absence of ulcers), and eight patients showed a response (defined as a SES-CD ≥ 50% improvement). Moreover, biochemical remission, defined as CRP ≤  5 mg/L, occurred in 50% of patients [42].

However, the study had several limitations that should be considered when interpreting the results. As a retrospective analysis, it is susceptible to recall bias and lacks the consistency of standardized data collection protocols. The steroid tapering process was left to the discretion of the investigators, which could have influenced the primary endpoint. Additionally, only a small proportion of patients underwent objective measures of inflammation, such as endoscopy, MRI, or IUS, which limits the ability to draw definitive conclusions about the long-term efficacy and safety of the treatment.

Favorable outcomes were observed in a prospective study, where risankizumab significantly reduced the HB index. Notably, positive progression could be seen as soon as week 2, with maximal reduction achieved by week 8, indicating a rapid and sustained therapeutic effect. After 12 weeks of observation, 78% of patients demonstrated a clinical response (≥3-point decrease in HB and/or HB < 5), with 70% achieving clinical remission (HB < 5), irrespective of previous treatment history with ustekinumab. However, patients who had not previously received ustekinumab showed significantly higher steroid-free clinical remission rates (72%) in comparison to those with prior ustekinumab exposure (55%, *p* = 0.003). Additionally, the median FCP levels showed a notable decrease, dropping from 395 at baseline to 114 at week 12, indicating a significant reduction in inflammation [43].

Previous studies have shown the superiority of risankizumab over placebo in treating moderate-to-severe Crohn’s disease. In addition, comparative trials with other biologics, such as ustekinumab, have also been conducted. The SEQUENCE trial, a phase 3b, randomized controlled study, involved 527 patients after inadequate response or intolerance to anti-TNF therapies. Participants were randomized to receive either risankizumab (255 patients) or ustekinumab (265 patients) over a 48-week period. In terms of clinical remission, defined as a CDAI score below 150, risankizumab demonstrated noninferiority to ustekinumab at week 24, with 58.6% of risankizumab-treated patients achieving remission compared to 39.5% in the ustekinumab group (AD 18.4% [95% CI] 6.6 to 30.3). Additionally, risankizumab showed endoscopic remission (SES-CD of ≤4 and ≥2-point reduction from baseline) at week 48, in 31.8% of patients compared to 16.2% in the ustekinumab group (AD 15.6% [95% CI] 8.4 to 22.9; *p* < 0.001) [44].

A recent matching-adjusted indirect comparison evaluating the efficacy of risankizumab and ustekinumab analyzed the data from different clinical trials. For risankizumab, this included the ADVANCE, MOTIVATE, and FORTIFY trials, while for ustekinumab, results obtained from the UNITI-1, UNITI-2, and IM-UNITI studies were analyzed. This comparison further confirmed the effectiveness of risankizumab. The drug achieved higher rates of clinical remission, with approximately 60.7% of patients reaching remission based on the CDAI (≤150) compared to ustekinumab’s 43.6% (*p* < 0.05). Additionally, risankizumab demonstrated a superior endoscopic response rate of 51.4%, which was significantly higher than ustekinumab’s 30.7% (*p* < 0.05) [45]. The treatment outcomes achieved with risankizumab are summarized in Table 1.

#### 3.1.3. Efficacy in Maintenance Therapy

Risankizumab has demonstrated its efficacy as a maintenance therapy as well. The FORTIFY trial, a phase 3, randomized, placebo-controlled study, evaluated risankizumab’s effectiveness in patients who previously showed a favorable response to induction therapy in the ADVANCE or MOTIVATE trials. A total of 542 participants were randomized into three groups: 180 mg risankizumab (179 patients), 360 mg risankizumab (179 patients), or placebo (184 patients). Treatment was administered subcutaneously every eight weeks for a duration of 52 weeks. The primary endpoints included clinical remission, defined as a CDAI score below 150 or SF ≤ 2.8 and AP ≤ 1, as well as endoscopic response, characterized by a reduction in SES-CD exceeding 50% compared to baseline [46].

The results demonstrated that the 360 mg dose of risankizumab led to markedly higher rates of clinical remission and endoscopic response in comparison to placebo at week 52. Specifically, 52% of patients in the 360 mg group reached CDAI clinical remission, versus 41% in the placebo group (95% CI 5–24, *p* = 0.0054). Additionally, SF/AP clinical remission was achieved by 52% of patients receiving the 360 mg dose compared to 40% in the placebo group (95% CI 5–25, *p* = 0.0037). Similarly, endoscopic response rates were significantly higher in the 360 mg group, with 47% of patients achieving this outcome versus 22% in the placebo group (95% CI 19–37, *p* < 0.0001) [46].

The 180 mg dose of risankizumab also yielded favorable results, showing superior rates of CDAI clinical remission and endoscopic response relative to placebo. In the 180 mg group, 55% of patients attained CDAI clinical remission (95% CI 5–24, *p* = 0.0031), and the endoscopic response rate was 47% (95% CI 17–35, *p* < 0.0001). However, the SF/AP clinical remission rates for the 180 mg dose did not show statistically significant differences when compared to placebo (*p* = 0.12) [46].

Comparing the two dosing regimens, the 360 mg dose demonstrated greater overall efficacy across key endpoints, including endoscopic remission, CDAI deep remission (defined as both CDAI and endoscopic remission), SF/AP deep remission (defined as both SF/AP and endoscopic remission), and the absence of ulcers in endoscopy. However, when the analysis was restricted to patients without previous biologic failure, the 180 mg dose was found to be more effective in this subgroup [46]. Furthermore, the FORTIFY trial highlighted the long-term benefits of achieving endoscopic response or remission following risankizumab induction. Patients who demonstrated endoscopic improvement after the 12-week induction phase and continued risankizumab maintenance therapy experienced significantly lower rates of CD-related hospitalizations and surgeries through week 52. Specifically, those achieving endoscopic response, remission, or ulcer-free endoscopy had significantly reduced hospitalization rates (1.7, 1.2, and 1.5 events per 100 person-years, respectively) compared to those who did not achieve these outcomes (6.4 events per 100 person-years, *p* < 0.05). Notably, no CD-related surgeries were reported among patients who attained endoscopic response, remission, or ulcer-free endoscopy, whereas surgery rates in those without these outcomes ranged from 2.4 to 3.2 events per 100 person-years (*p* = 0.025). In contrast, patients assigned to placebo during maintenance exhibited no significant difference in hospitalization or surgery rates, regardless of their initial endoscopic response. These findings suggest that early endoscopic improvement with risankizumab correlates with better long-term disease control [47].

Data from the ADVANCE, MOTIVATE, and FORTIFY studies were used by Peyrin-Biroulet et al. [48] to assess whether the CD duration is linked with risankizumab effectiveness, both in the induction and maintenance phases. The patients were divided into four groups depending on CD duration (under 2 years, 2 to 5 years, more than 5 to 10 years, and more than 10 years). After 12 weeks, patients treated with risankizumab who had shorter CD durations had significantly higher response rates for endoscopic response (48.3%, 36.3%, 32.0%, and 33.4%, respectively; *p* = 0.025), endoscopic remission (34.9%, 23.5%, 17.1%, and 20.3%, respectively; *p* = 0.009), and ulcer-free endoscopy ([0 in SES-CD ulcerated surface subscore] 30%, 19.7%, 17.8%, and 13.1%, respectively; *p* = 0.001). However, while SF/APS clinical remission also showed higher response rates in patients with shorter CD duration (42.7%, 46.9%, 43.5%, 33.2%, respectively, *p* = 0.046), CDAI clinical response and remission and enhanced clinical response did not show any significant difference between the age groups. At week 52, patients from the FORTIFY study treated with 180 mg risankizumab dose who had shorter CD durations had significantly higher response rates for SF/APS and CDAI clinical remission (SF/APS: 56.0%, 62.5%, 41.0%, 37.7%, respectively, *p* = 0.027; CDAI: 68%; 65%, 56.4%, 44.3%, respectively, *p* < 0.017), endoscopic remission (52.0%, 31.3%, 25.6%, 23.0%, respectively, *p* = 0.013), and ulcer-free endoscopy (40.0%, 28.1%, 17.9%, 19.7%, respectively, *p* = 0.044), although surprisingly there were no significant differences between the age groups regarding endoscopic response. In the 360 mg risankizumab group, no significant differences existed between the age groups in any of the criteria. During both induction and maintenance phases, the total incidence of TEAEs and TEAEs of safety interest was comparable regardless of the age group.

Patients from the ADVANCE and MOTIVATE studies who did not achieve SF/APS clinical remission during the twelve weeks of treatment could receive another 12 weeks of risankizumab therapy in the double-blind study by Panacione et al. [49] In the study, 252 out of 313 eligible patients were rerandomized 1:1:1 to receive 180 mg or 360 mg of risankizumab subcutaneously, or 1200 mg of risankizumab intravenously. SF/APS clinical response rates for patients not responding to initial 12-week treatment, at week 24 of prolonged risankizumab therapy were as follows: 62.3% with 1200 mg intravenous group (95% CI, 51.5–73.2%), 76.2% with 180 mg subcutaneous group (95% CI, 67.0–85.3%), and 63.7% with 360 mg subcutaneous group (95% CI, 53.9–73.6%). The patients who achieved clinical response at week 24 were eligible to enter the FORTIFY trial and continued treatment to week 52. The 180 mg and 360 mg group continued their treatment, while the patients from the 1200 mg group who achieved clinical response were again rerandomized 1:1:1 to receive either placebo, 180 mg, or 360 mg subcutaneously. The majority of patients treated with risankizumab subcutaneously with delayed SF/APS clinical response continued to show clinical response at week 52; however, a group with a higher dose had a better response (69.7% in the 360 mg group compared to 56.7% in the 180 mg group).

Zare et al. conducted a retrospective cohort study across two tertiary IBD centers in the UK, confirming the real-world efficacy of risankizumab as both induction and maintenance treatment for patients with active CD. The trial included 53 patients who exhibited symptoms and/or signs of active inflammation, such as an increase in CRP, and endoscopic or radiologic evidence of disease activity. All participants had previously not responded adequately or were intolerant to biologic therapies, including infliximab, adalimumab, vedolizumab, and ustekinumab [50].

Patients received intravenously 600 mg of risankizumab at weeks 0, 4, and 8 as induction therapy, with subsequent subcutaneously administered maintenance dosing in 8-week intervals starting at week 12. Among these, 45 patients received 360 mg maintenance dosing, while eight patients initiated treatment with 180 mg, with six of them subsequently escalated to 360 mg based on clinical assessments [50].

The study assessed clinical response, which was defined as a ≥3-point reduction in HB or achieving HB < 5. At week 12, 33% of patients achieved clinical response, which increased to 45% at week 28 and 52% at week 52. Clinical remission (HB < 5) was observed in 31%, 40%, and 44% of patients at weeks 12, 28, and 52, respectively. Notably, among patients without initial response or remission by week 12, seven individuals achieved a delayed clinical response later in the study period, with most also meeting the criteria for clinical remission [50].

Biochemical remission, characterized by CRP ≤ 5 mg/L or FC ≤ 200 µg/g, demonstrated gradual improvements. CRP remission was achieved in 24% at week 12 and 36% at weeks 28 and 52, while FC remission rates were 25%, 17%, and 38% at weeks 12, 28, and 52, respectively. Combined biochemical remission (CRP ≤ 5 mg/L and FC ≤ 200 µg/g) was not observed at week 12 but was achieved in 16% of patients at both weeks 28 and 52 [50].

Among patients receiving corticosteroids at baseline (34%, *n* = 18), 67% discontinued steroid use within 12 weeks of risankizumab treatment, and a further 22% discontinued during maintenance therapy. By week 52, corticosteroid-free remission was achieved in 29% at week 12, 37% at week 28, and 44% at week 52 [50].

After a follow-up period of 515 days (median), risankizumab exhibited high efficacy, with 74% of patients remaining on therapy [50]. 

Risankizumab also exhibited sustained clinical effectiveness through weeks 26 and 52 in patients with refractory Crohn’s disease, as demonstrated by real-world data from the GETAID study. At week 26, 47% of patients achieved steroid-free clinical remission, while 52% reached clinical remission based on the HB. By week 52, these rates remained stable at 46% and 48%, respectively. Among patients with high disease activity at baseline (HB ≥ 8), 38% achieved steroid-free clinical remission by week 52, and 87.9% of those using steroids at baseline were able to discontinue them. Extraintestinal manifestations also improved, with 15 of 35 patients achieving complete remission. Among those with active perianal Crohn’s disease, remission and response rates were each 33%. Hospitalization and intestinal resection were required in 20.9% and 12.6% of patients, respectively [51]. 

Inflammatory biomarkers showed significant reductions over time. In patients with objective signs of inflammation at baseline, 47%, 43%, and 45% achieved steroid-free clinical remission at weeks 12, 26, and 52, respectively. Median CRP levels decreased from 8 mg/L at baseline to 6 mg/L at week 12 and 4 mg/L at week 52 (*p* = 0.02), while fecal calprotectin levels showed a significant median reduction of 592 mg/kg (*p* = 0.001). Endoscopic or radiologic assessments in 79 patients revealed remission in 22% and response in 34% [51].

Risankizumab persistence rates were high; however, 26% of patients discontinued treatment during follow-up, primarily due to loss of response (42%) and primary non-response (37%) [51].

When evaluating the efficacy of risankizumab and ustekinumab during the maintenance phase, CDAI remission rates were notably similar, ranging from 59 to 61.1% for risankizumab and 53.1% for ustekinumab, showing no statistically significant differences. However, risankizumab demonstrated a greater outcome in terms of mucosal healing, with significantly higher rates of endoscopic response (60.7% versus 24.1%) and endoscopic remission (29.5% versus 10.9%) compared to ustekinumab, regardless of the dosing regimen employed. These findings suggest that while both medications may achieve comparable clinical remission, risankizumab proved to be more effective in promoting mucosal healing [45].

#### 3.1.4. Adverse Effects

The safety profile of risankizumab has been extensively studied in several large-scale clinical trials. Across these studies, the incidence of treatment-related adverse events was generally consistent between the 180 mg and 360 mg dose groups, with no evidence of dose-dependent safety concerns. The overall incidence of adverse events was 48–59%, while serious ones occurred in approximately 5% of patients [40]. The most frequently reported adverse events among patients receiving risankizumab, which are listed in Table 2, included nasopharyngitis, arthralgia, headache, abdominal pain, and nausea, affecting up to 9% of participants. In contrast, the most common adverse reaction in groups treated with placebo was exacerbation of CD, which impacted up to 16% of patients, despite similar baseline disease activity (assessed by CDAI and SES-CD values) across randomized groups [40,46]. This likely occurred due to the lack of any active therapeutic effects from the placebo.

Injection site reactions were mild to moderate and occurred at slightly higher rates (1–6%) among patients receiving 360 mg relative to the 180 mg (1–5%) and placebo (1–5%) groups [40,46]. Other reported minor adverse events included fatigue, pruritus, paresthesia, and upper respiratory tract infections, documented in as many as 12% of smaller studies’ patients [43].

The incidence of serious adverse events was lower with risankizumab (10.3%) compared to ustekinumab (17.4%), with both drugs showing similar rates of serious infections (3.1% and 4.2%, respectively) and hepatic events (6.9% and 5.3%, respectively) [44]. In placebo-controlled trials, most serious adverse events were related to underlying CD rather than the drug. Rare cases of appendicitis, pneumonia, urinary tract infection, viral pharyngitis, and sepsis were reported but considered not to have any link to risankizumab [40,46]. In the GETAID study, 7% of patients experienced serious adverse events, including CD exacerbations and hypertensive flare-ups requiring hospitalization. None of the events mentioned resulted in death [42].

No deaths were reported during the maintenance phase with risankizumab treatment in the FORTIFY trial [46]. In contrast, two deaths occurred in the placebo group during the ADVANCE trial, and one death was reported in the 1200 mg risankizumab group in the MOTIVATE trial. However, the latter was deemed unrelated to the study drug [40]. Additionally, no treatment-related malignancies were observed, apart from a single case of HER-2-positive breast cancer in the 360 mg risankizumab group, assessed as unrelated to the medication [40,42,46].

Patients treated with risankizumab exhibited a slightly higher incidence of herpes zoster compared to the placebo group (three cases versus one case). However, these cases occurred in patients who were also receiving concomitant corticosteroid or azathioprine therapy, which may have contributed to the increased risk. All cases were mild and resolved without discontinuation of the drug [40]. Rare opportunistic infections such as oral candidiasis and intestinal *Aeromonas* infection occurred in two patients and were self-limiting [46]. A single case of tuberculosis was noted in a patient receiving risankizumab who had previously experienced active tuberculosis [40].

Transient elevations in liver enzymes were generally observed in less than 3% of participants across studies [40,46] and reached up to 6.9% in the study by Peyrin-Biroulet et al. [44]. No severe or serious liver-related events were recorded across all studies. One hypersensitivity reaction involving a rash and liver enzyme elevation occurred; however, it resolved with steroids and there was no evidence of drug-induced liver injury [40].

Compared to ustekinumab, risankizumab demonstrated a lower incidence of serious adverse events and infections and a slightly higher incidence of hepatic events [44]. The risk of malignancies, hypersensitivity, and infusion reactions was low across all studies.

In conclusion, risankizumab demonstrates a positive safety record characterized by a low frequency of serious adverse reactions and no treatment-related deaths or malignancies. These findings, combined with its efficacy, position risankizumab as a well-tolerated and effective therapeutic option for managing inflammatory bowel disease, particularly for patients requiring advanced treatment strategies.

### 3.2. Guselkumab

#### 3.2.1. General Information

Guselkumab is a human IgG1λ monoclonal antibody specifically targeting the p19 component of IL-23 [52]. Initially approved in 2017 for moderately to severely active plaque psoriasis and later for active psoriatic arthritis, recent advancements suggest its potential application in treating moderate-to-severe Crohn’s disease [53]. The GALAXI program (Phase 2/3 studies) has been instrumental in evaluating guselkumab’s efficacy and safety for CD. Results from the GALAXI 2 and 3 trials demonstrated superior outcomes compared to placebo and ustekinumab, including significant improvements in clinical remission and endoscopic response. These findings underscore guselkumab’s potential as an innovative therapeutic option for managing CD [54].

#### 3.2.2. Efficacy in Induction Therapy

In two phase 2 trials, including patients with moderately to severely active CD, guselkumab exhibited substantial effectiveness, even in those who had been refractory or intolerant to prior biologic or conventional therapies [55].

In these placebo-controlled studies, participants were administered guselkumab intravenously at dosages of 200 mg, 600 mg, or 1200 mg. By week 12, a significant proportion of patients achieved clinical remission based on the CDAI and improvements in patient-reported outcomes, such as stool frequency and abdominal pain [56,57]. GALAXI 2 primarily assessed the efficacy of guselkumab over a 48-week period, focusing on clinical remission (CDAI < 150) and endoscopic response rates during both induction and maintenance therapy. The study provided early insights into the short-term effectiveness of guselkumab in inducing remission and achieving endoscopic improvements [54,55].

GALAXI 3, in contrast, emphasized long-term safety and efficacy, particularly in patients with a history of treatment failure with other biologics. It also evaluated the durability of response across different dosing regimens over extended periods, making it particularly valuable for understanding guselkumab’s role in complex cases of CD [54,55].

In the GALAXI trials, guselkumab demonstrated significant clinical and endoscopic efficacy in patients with moderate-to-severe CD. Clinical remission, characterized by a Crohn’s Disease Activity Index score of <150, was attained by 40–50% of patients receiving guselkumab depending on the dose, in comparison to 20% in the placebo group [56]. Endoscopic improvement was measured using SES-CD, a standardized tool assessing mucosal healing. Endoscopic mucosal healing (characterized by SES-CD ≤ 4 and ≥2-point reduction from baseline with no subscore > 1 in any component) was achieved by 35–45% of patients treated with guselkumab, in comparison to only 15% in the placebo group [55].

Additionally, significant reductions in biomarkers of inflammation, such as CRP and fecal calprotectin, were demonstrated. At week 4, median CRP levels decreased by 3.1 mg/L (±2.5) in the guselkumab group compared to 0.5 mg/L (±1.1) in the placebo group. By week 12, these reductions were sustained, with median CRP levels dropping further to 4.3 mg/L (±3.6) for guselkumab versus 1.2 mg/L (±0.9) in placebo-treated patients. Similarly, fecal calprotectin, a biomarker of intestinal inflammation, showed a median decrease of 200 μg/g (±125) at week 4 in guselkumab-treated patients, compared to only 50 μg/g (±20) in the placebo group. By week 12, these values continued to decline, with guselkumab demonstrating reductions of approximately 250–300 μg/g (±150), while the placebo group showed only modest changes [58].

Similarly, in the real-world cohort study involving highly refractory CD patients, guselkumab showed a clinical response in nearly 70% of participants. These patients had severe disease, often characterized by a history of multiple biologic failures, including anti-TNF agents and ustekinumab. Specifically, the study population included patients with moderately to severely active CD, characterized by a CDAI score of ≥220, high levels of inflammation biomarkers (elevated CRP and fecal calprotectin), and/or persistent symptoms like abdominal pain and diarrhea despite previous therapies [55,59,60]. The cohort consisted of individuals with advanced disease features, such as penetrating or stricturing disease under the Montreal classification (classifies the severity of Crohn’s disease), and many had experienced secondary non-response to prior biologics. This highlights guselkumab’s potential efficacy in addressing treatment-resistant CD cases [61].

These findings suggest that guselkumab is an effective induction therapy for Crohn’s disease, offering a promising option for patients with difficult-to-treat disease or those who have failed other biologic treatments. The treatment outcomes achieved with guselkumab are summarized in Table 3.

#### 3.2.3. Efficacy in Maintenance Therapy

Guselkumab has also shown significant efficacy as a maintenance therapy in CD. In a phase 2, randomized, placebo-controlled trial, patients who initially responded to guselkumab during induction therapy were enrolled in a maintenance phase to assess the drug’s long-term effectiveness. Participants were randomly assigned to undergo subcutaneous guselkumab administration at doses of 100 mg or 200 mg every eight weeks, or placebo, for up to 48 weeks. The primary endpoints included clinical remission, measured by the CDAI, and endoscopic response, evaluated using the SES-CD [55,61].

The results demonstrated that guselkumab led to sustained clinical remission and significant endoscopic improvement. By week 48, 55–60% of patients receiving guselkumab maintained CDAI clinical remission. This was notably superior to the 27% remission rate observed in the placebo group, highlighting the efficacy of guselkumab for those with refractory CD in maintaining low disease activity and improving patient outcomes over nearly a year of treatment. Endoscopic response rates were similarly favorable, with 45% of patients in the guselkumab 200 mg group achieving mucosal healing compared to only 18% in the placebo group. This difference, as noted with the 95% CI of 15–32, emphasizes the statistical significance of guselkumab’s impact on endoscopic improvement. Clinical remission based on PRO-2, such as stool frequency and abdominal pain, was observed in 50–55% of patients treated with guselkumab, significantly higher than the 30% observed in the placebo group [55,63,64]. The assessment of abdominal pain was measured using a 0 to 10 scale, where 0 signifies no pain at all, and 10 represents the most severe imaginable pain. Patients rated their abdominal pain daily on this scale, providing a direct and subjective measure of symptom severity [55,64].

Guselkumab also demonstrated reductions in biomarkers of inflammation, including CRP and fecal calprotectin, throughout the 48-week maintenance period. These reductions were more pronounced in the 200 mg group, where CRP levels decreased by over 60%, and fecal calprotectin dropped by 50%, confirming its ability to sustain both clinical and biochemical remission in CD [65].

In a real-world comparison with ustekinumab, guselkumab demonstrated comparable CDAI clinical remission rates, but showed superior efficacy in achieving endoscopic response and mucosal healing. This suggests that while both therapies are effective in maintaining remission, guselkumab may offer additional benefits in terms of long-term intestinal healing [60,61,66].

#### 3.2.4. Special Advantages and Disadvantages Compared to Previous Therapies

Compared to ustekinumab and other existing biologics, guselkumab has demonstrated greater efficacy in achieving mucosal healing, an important indicator of long-term intestinal health. Additionally, guselkumab’s dosing schedule of every eight weeks may be more convenient for some patients compared to therapies requiring more frequent administration. However, its subcutaneous injection form might be a drawback for patients who prefer oral tablets, as seen with emerging small-molecule therapies like JAK inhibitors.

One disadvantage noted by some patients is the perceived discomfort of injections, although this is common with biologics. On the other hand, patients report high satisfaction with the consistent clinical and endoscopic outcomes achieved with guselkumab, particularly when refractory to other treatments [55,60,64].

#### 3.2.5. Patient Opinions and Preferences

Discussions around guselkumab have highlighted a division in patient preferences. While many appreciate the reduced dosing frequency and improved clinical outcomes, others express a preference for oral medications due to ease of use and avoidance of injections. Some patients, especially those new to biologic therapy, may feel apprehensive about the injection process, though most adapt over time [55,64,65].

Overall, guselkumab’s long-term benefits in maintaining remission, reducing inflammation, and promoting intestinal healing position it as a highly effective option, though patient preferences regarding delivery methods should be carefully considered in clinical decision-making.

#### 3.2.6. Adverse Effects and Limitations

The safety profile of guselkumab in Crohn’s disease has been evaluated in the clinical trials mentioned, demonstrating a favorable risk-to-benefit ratio. In a phase 2 trial, guselkumab showed favorable tolerability, with the most reported adverse effects being mild to moderate, including headache, fatigue, and upper respiratory tract infections. Serious adverse events were reported in less than 5% of patients, with no deaths or treatment-related malignancies observed during the study [55]. The most common adverse effects are presented in Table 4.

Despite its promising efficacy, guselkumab has several limitations in the treatment of Crohn’s disease. One major concern is the limited clinical data, as its use in Crohn’s disease is primarily supported by phase 2 trials, with a lack of large-scale phase 3 trials and long-term safety data [67]. Additionally, guselkumab may have a delayed onset of action compared to TNF inhibitors, which could be a disadvantage for patients requiring rapid symptom relief. Another limitation is the potential for secondary loss of response over time, necessitating long-term monitoring and possible dose adjustments. While guselkumab has a favorable safety profile, its mechanism of action in modulating the immune system may increase the risk of opportunistic infections. Moreover, there is a lack of head-to-head comparative data with other IL-23 inhibitors, making it difficult to establish its relative efficacy. Finally, cost and accessibility remain challenges, as newer biologics like guselkumab can be expensive and may not be widely available in all healthcare systems, limiting patient access. Despite these challenges, ongoing research is needed to further define its long-term role in Crohn’s disease management and optimize its use in clinical practice.

Similarly, in a real-world cohort study [61], the safety record of guselkumab aligned with results reported in prior clinical studies [54,55]. In the study, 15% of patients experienced adverse events, primarily consisting of mild infections such as nasopharyngitis and sinusitis. Serious adverse events occurred in two of the patients, who experienced severe infections. No cases of active tuberculosis, opportunistic infections, or fatalities were reported [55].

In long-term maintenance therapy, the rates of adverse effects remained low. A phase 2 extension study showed that common side effects included headache, arthralgia, and injection site reactions, while serious infections such as pneumonia were rare, affecting less than 1% of patients receiving guselkumab. Importantly, no significant increase in hepatic events or cardiovascular issues was reported during the study [55,68].

In comparison to ustekinumab, guselkumab demonstrated a similar or slightly lower incidence of serious adverse events—nasopharyngitis (25 [11%] of 220 guselkumab recipients, 12 [11%] of 114 ustekinumab recipients) and upper respiratory infections (13 [6%] guselkumab recipients, eight [7%] ustekinumab recipients). After week 12, one patient who showed a response to placebo induction and two patients receiving guselkumab experienced severe infections. No instances of active tuberculosis, opportunistic infections, or fatalities were reported [54,55].

The rates of serious infections and hospitalizations due to Crohn’s disease exacerbation were comparable between the two therapies, indicating a consistent safety profile across different biologic agents. These results provide additional evidence supporting the use of guselkumab as a well-tolerated treatment for patients with moderate to severe Crohn’s disease [69].

Guselkumab, an IL-23 inhibitor, demonstrates a low rate of serious adverse events (SAEs) in CD treatment in comparison to other biologics. Results from the GALAXI studies (Phase 2 and ongoing Phase 3 trials) indicate that guselkumab’s safety profile is consistent and favorable across both induction and maintenance therapy phases, with a low incidence of serious infections and no reported cases of malignancies or mortality in the studies analyzed. These findings align with the safety data from its use in psoriasis and psoriatic arthritis [53,54,70].

When compared to other biologics like ustekinumab, guselkumab appears equally safe or slightly better in certain metrics, such as the rates of infections and malignancies. For ustekinumab, pooled long-term safety data also reported no increased malignancy risk, but guselkumab has shown stability in adverse event rates even during prolonged therapy [71].

This favorable safety profile, along with its sustained efficacy, positions guselkumab as a promising treatment option for Crohn’s disease. Ongoing studies will further clarify its place relative to other biologics.

### 3.3. Mirikizumab

#### 3.3.1. General Information

Mirikizumab is a humanized IgG4 anti-human monoclonal antibody directed against the p19 component of IL-23 [72]. In May 2023, the European Medicines Agency authorized the use of mirikizumab as an induction and maintenance treatment for moderate to severe ulcerative colitis [73] and later in October, it was also approved by the FDA for treating patients with the same condition [74].

#### 3.3.2. Efficacy in Induction and Maintenance Therapy

The effectiveness of mirikizumab as an induction therapy was studied in 2022 by Sands BE et al. [75] In this phase 2, randomized, multicenter, 52-week trial, 191 patients with moderately to severely active CD (SF ≥ 4, daily abdominal pain [AP] ≥ 2, SES-CD ≥ 7 in ileocolic CD or 4 ≥ in ileal CD) were studied. All patients had previously received aminosalicylates, 6-mercaptopurine, azathioprine, or corticosteroids, and/or at least one biologic agent (anti-TNF, vedolizumab, or experimental) excluding those targeting IL-23 p19 with either inadequate or lack of response or who were corticosteroid-dependent.

The patients were randomized into four groups (2:1:1:2) to be administered either placebo, 200 mg, 600 mg, or 1000 mg mirikizumab intravenously every 4 weeks until week 12. Then all patients who were administered mirikizumab and showed progress (1 ≥ point decrease in SES-CD score from baseline) were rerandomized evenly to either continue receiving the same dose of intravenous mirikizumab from the induction phase with subcutaneous placebo every 4 weeks (IV-C) or receive a placebo intravenously and 300 mg of mirikizumab subcutaneously (IV/SC). The patients who received a placebo from the start of the trial as well as those who did not experience improvement were given 1000 mg of mirikizumab plus a subcutaneous placebo every 4 weeks.

At week 12 since the beginning of the trial, the endoscopic response (a 50% reduction in baseline SES-CD) in the 600 mg and 1000 mg groups was significantly higher compared to the placebo group (37.5% for the 600 mg group (*p* < 0.01), and 43.8% for the 1000 mg group (*p* < 0.001) compared to 10.9% in placebo). The same is true for endoscopic remission (score of <4 for ileal-colonic disease or <2 for isolated ileal disease and no subscore > 1; *p* < 0.01). However, each of the three groups treated with mirikizumab had a significant change in quality of life with the Inflammatory Bowel Disease Questionnaire (IBDQ) score (*p* < 0.001), and significantly decreased SF, and AP from baseline. Patients treated with the 1000 mg dose had the greatest reductions in high-sensitivity CRP (a sensitive test for measuring CRP, which can measure even the slightest levels of CRP in plasma; *p* < 0.001) and FCP levels (*p* < 0.001) compared to baseline. Patients treated with the 600 mg dose had better Patient Reported Outcomes (PROs) response (reduction ≥ 30% in SF and/or AP and no deterioration from baseline; *p* < 0.01) and remission (SF ≤ 2.5 and AP ≤ 1; *p* < 0.05) and CDAI response (CDAI < 150 or decrease ≥ 100 points from baseline; *p* < 0.01) and remission (CDAI < 150; *p* < 0.001).

The efficacy of mirikizumab was also tested in the VIVID-1 trial [76]. In this phase 3, randomized, double-blind, placebo-controlled study, 1065 adults with moderate to severe CD (SF ≥ 4 and/or AP ≥ 2 at baseline, SES-CD ≥ 7 in ileocolic CD or 4 ≥ in ileal CD within 21 days before randomization) were randomized in three groups 6:3:2. The first group was administered 900 mg of mirikizumab intravenously at 4-week intervals until week 12, then 300 mg subcutaneously at 4-week intervals until week 52; the second one was administered 6 mg/kg IV of ustekinumab once, then 90 mg SC every 8 weeks; and the third group was placebo. At week 52 out of all 579 patients treated with mirikizumab, 48.4% had an endoscopic response (*p* < 0.0001) and 54.1% had clinical remission with CDAI (*p* = 0.0014), compared to the placebo group’s 9% and 19.6%, respectively. Mirikizumab also performed better in endoscopic response and corticosteroid-free CDAI remission compared to placebo (32.5% and 43.7% in the mirikizumab group compared to 12.6% and 18.6% in the placebo group, respectively; *p* < 0.0001). In comparison to ustekinumab, mirikizumab achieved non-inferiority for CDAI clinical remission. However, the superiority of mirikizumab compared to ustekinumab for the endoscopic response was not achieved. The treatment outcomes achieved with mirikizumab are summarized in Table 5.

The study, however, has some limitations. The most prominent limitation is the incorporation of the placebo non-responders into the mirikizumab group after week twelve, which can affect the interpretation of the results. Another drawback is that the trial did not evaluate the effectiveness and safety of intravenous rescue therapy or prolonged intravenous induction in patients who either had a poor response or who first reacted but then stopped responding.

#### 3.3.3. Adverse Effects

The safety profile of mirikizumab has also been studied by Sands BE et al. in patients with CD. During the indication phase, treatment-emergent adverse effects (TEAEs) occurred in 58.1% of patients in the 200 mg mirikizumab group, 65.6% in the 600 mg and 1000 mg groups, and were higher in the placebo group (70.3%); they are presented in Table 6. The most common adverse effects of mirikizumab include headaches, worsening of CD, arthralgia, nasopharyngitis, increase in body weight, anemia, and nausea. The incidence of those adverse effects does not show a dose-dependent correlation. Out of all 127 patients treated with mirikizumab, only five had at least one serious adverse event. Those were chest pain, worsening of CD, colon stenosis, colon perforation (in the 600 mg group), and abdominal and back pain (in the 1000 mg group). During the maintenance period, TEAEs occurred in 75.6% of IV-C patients and 76.1% of IV/SC patients, the most common being nasopharyngitis, headache, arthralgia, anemia, and injection site pain. The SAEs occurred only in two patients in the IV/SC group. One patient experienced ileal perforation and peritonitis, while the other experienced worsening of CD, pyelonephritis, and dehydration. There were no instances of death or malignancy either in the induction or maintenance phase [75]. In another study, the VIVID-1 trial, the TEAEs occurred with a frequency of 78.6% [76]. In the study, COVID-19, anemia, headache, arthralgia, and respiratory tract infections were the most frequently observed adverse events. No deaths were reported in the patients treated with mirikizumab; however, two patients developed malignant lesions—one breast cancer and one basal cell carcinoma.

#### 3.3.4. Comparison of Risankizumab, Guselkumab, and Mirikizumab in the Treatment of CD

Risankizumab, guselkumab, and mirikizumab have similar safety profiles, with mild to moderate adverse effects like nasopharyngitis, headache, arthralgia, and injection site reactions. Serious adverse events are low (<5%) for all three, with serious infections occurring in 2–3% of patients. Risankizumab has the most extensive data, with 10.3% SAE incidence, but no treatment-related deaths or malignancies. Guselkumab shows comparable safety, with serious infections < 2%, while mirikizumab has a slightly higher overall adverse event rate (75–78%), including two malignancies, though causality is unclear.

In terms of remission duration, risankizumab maintains remission in ~52% of patients at 52 weeks, guselkumab in 55–60% at 48 weeks, and mirikizumab in ~54% at 52 weeks, suggesting similar long-term efficacy. Risankizumab has the strongest clinical backing, guselkumab shows promising mucosal healing, and mirikizumab may be an alternative for non-responders to other biologics. Further studies are needed to confirm long-term differences.

Overall, all three IL-23 inhibitors provide a strong safety profile with low rates of serious complications, and their efficacy in maintaining long-term remission appears comparable. However, risankizumab has the most robust long-term data, guselkumab shows promising mucosal healing benefits, and mirikizumab may serve as an alternative option for patients not responding to other biologics. Further head-to-head studies are needed to determine the optimal positioning of these drugs in Crohn’s disease management.

## 4. IL 23 Inhibitors Versus Other Biologic Drugs in CD

Biologic therapies play a critical role in managing moderate-to-severe Crohn’s disease. TNF antagonists, such as infliximab and adalimumab, are recommended as first-line biological agents in patients who have not responded to conventional therapies [68]. These therapeutics, particularly in combination with azathioprine, remain highly effective as first-line therapies for biologic-naive patients, demonstrating efficacy for the induction (infliximab’s OR 6.55 [95% CI: 2.31, 18.55] and adalimumab’s OR 5.31 [95% CI: 2.99, 9.43]) and maintenance of clinical remission. However, their effectiveness is limited in patients who have failed to respond to TNF antagonists, a population increasingly common in clinical practice. For these patients, IL-23 blockades with risankizumab or ustekinumab offer a promising alternative. Both agents have shown efficacy in inducing (risankizumab’s OR 2.64 [95% CI: 1.89, 3.68] and ustekinumab’s OR 2.55 [95% CI: 1.39, 4.69]) and maintaining remission after TNF failure, with risankizumab demonstrating superiority over vedolizumab for the induction of remission in network meta-analyses. Additionally, risankizumab outperformed ustekinumab in endoscopic response and remission in key trials, emphasizing its role in achieving long-term disease control [77]. Since endoscopic remission is associated with several clinical benefits, risankizumab treatment may reduce hospitalizations, reliance on corticosteroids, and the risk of developing colorectal dysplasia in patients. Another advantage of risankizumab is its targeted mechanism of action against the IL-23p19 subunit, which specifically addresses molecular pathways associated with resistance to TNF antagonists. While TNF inhibitors remain pivotal for biologic-naive patients, IL-23 inhibitors, particularly risankizumab, are emerging as a preferred option for patients with refractory or biologic-exposed CD, offering efficacy across a broader spectrum of disease severity.

As IL-23 p19 subunit inhibitors are a fairly new addition to the treatment of CD, with the first study being published in 2021, research comparing the efficacy and safety profile of these drugs to other biological agents in CD treatment is scarce. At the moment of writing this article, no head-to-head studies comparing the effectiveness of IL-23 p19 subunit inhibitors in CD to TNF inhibitors or vedolizumab have been published. Also, no head-to-head studies comparing the effectiveness of IL-23p19 subunit inhibitors to each other have been published.

In 2024, Moćko et al. [78] compared the effectiveness and safety profile of mirikizumab to other biological drugs (including adalimumab, ustekinumab, golimumab, infliximab, and vedolizumab), in moderate to severe UC therapy. In this systematic review with frequentist network meta-analysis, 14 randomized controlled trials were selected. The analysis showed no significant differences between mirikizumab and other biologic drugs in achieving clinical response and remission in the induction and maintenance phases. Also, no significant differences were noted in adverse events, infections, or SAEs between mirikizumab and other biological drugs. However, mirikizumab was linked to a decreased probability of treatment withdrawal due to adverse events during the induction phase compared to adalimumab (OR 0.21; 95% CI 0.11–0.4) and vedolizumab (OR 0.1; 95% CI 0.02–0.55).

Also in 2024, Dignass et al. [79] compared the effectiveness and safety profile of mirikizumab in induction and maintenance therapy to other biological drugs (including adalimumab, filgotinib, golimumab, infliximab, ozanimod, tofacitinib, upadacitinib, ustekinumab, and vedolizumab) in treating moderate to severe UC. This systematic review and network meta-analysis compared the efficacy and safety in biologic/Janus kinase inhibitors-naive and biologic/JAK inhibitors-experienced populations. In induction therapy in the experienced group, mirikizumab was significantly better than adalimumab in both clinical response and remission, and inferior to upadacitinib. In induction therapy in the naive group and in the maintenance therapy in the experienced group, it was also inferior to upadacitinib both in clinical response and remission. However, mirikizumab showed significantly better clinical response and remission in maintenance therapy in the naive population, compared to nearly all other biological drugs, except for ustekinumab and filgotinib. Additionally, compared to other biological drugs, mirikizumab did not show any significant increase or decrease in SAEs occurrence; however, it did show a reduction in all-cause treatment discontinuation compared to adalimumab (OR 0.29; 95% CI 0.14–0.59) and tofacitinib (OR 0.48; 95% CI 0.24–0.93).

In 2023, Kridin et al. [80] compared in a global population-based cohort the probability of complications of infections in psoriatic patients treated with IL-23 inhibitors (risankizumab, guselkumab, and tildrakizumab) and in those treated with TNF inhibitors (adalimumab, infliximab, etanercept, infliximab and certolizumab pegol). Compared to TNF inhibitors, IL-23 inhibitors showed a significantly decreased risk of certain diseases, including encephalitis (HR 0.18; 95% CI 0.04–0.78), hepatitis B reactivation (HR 0.24; 95% CI 0.12–0.47), cytomegalovirus (HR 0.25; 95% CI 0.07–0.86), influenza (HR 0.52; 95% CI 0.38–0.71), herpes zoster (HR 0.58; 95% CI 0.41–0.82), otitis media (HR 0.66; 95% CI 0.44–0.97), and parasitic disease (HR 0.78; 95% CI 0.64–0.95).

In the same year, Krueger et al. [81] assessed the risk of malignancies in patients receiving IL-23 inhibitors (risankizumab, guselkumab, and tildrakizumab) compared to those taking TNF inhibitors (adalimumab, infliximab, etanercept, and infliximab and certolizumab pegol), during the first five years of starting the treatment of psoriasis. In total, 5832 patients initiating IL 23 inhibitors and 5832 initiating TNF inhibitors were compared. The risk of malignancies was evaluated within two years and between two to five years after the start of treatment, to stratify by time the risk of malignancy. Compared to TNF inhibitors, IL-23 inhibitors showed a significantly smaller risk of certain malignancies through five-year follow-up time, including non-Hodgkin lymphoma (0.8 vs. 0.2%), hepatobiliary cancer (3.3% vs. 0.7%), and basal cell cancer (3.3% vs. 1.4%). The risk of malignancies was also compared with biologic-naive patients in which IL-23 inhibitors showed a significantly smaller risk of colorectal cancer (1.1% vs. 0.3%), basal cell carcinoma (3.4% vs. 1.5%), and Cutaneous squamous cell carcinoma (0.6% vs. 0.2%) during 5-year follow-up.

Guselkumab has also shown potential advantages over older biologicals like TNF inhibitors (e.g., infliximab, adalimumab) in Crohn’s disease. While TNF inhibitors remain effective as first-line biologics, particularly for induction and maintenance in biologic-naive patients, guselkumab offers a targeted mechanism of action that specifically addresses pathways associated with resistance to TNF therapies. Compared to ustekinumab, guselkumab has demonstrated comparable efficacy in achieving clinical remission but showed superior results in endoscopic healing frequency [60,61,65]. Moreover, guselkumab has been associated with a lower risk of infections and certain malignancies, such as basal cell carcinoma and hepatobiliary cancers, compared to TNF inhibitors [80,81]. These findings suggest guselkumab may be particularly beneficial for patients with refractory or biologic-experienced Crohn’s disease, offering improved outcomes in both clinical and safety profiles.

Recent advances in IL-23 inhibitors, including risankizumab, mirikizumab, and guselkumab, highlight their advantages over older treatments like TNF inhibitors for patients previously exposed to biological treatments or with refractory Crohn’s disease. These agents not only provide improved efficacy in achieving clinical remission and endoscopic healing but also exhibit a better safety profile, with a reduced risk of infections and certain malignancies. Their targeted mechanisms of action address molecular pathways associated with resistance to TNF therapies, making them a promising option for long-term disease management.

## 5. Future Prospects of Specific IL-23 Antagonists in CD Treatment

### 5.1. Brazikumab

Brazikumab, a humanized IgG2 monoclonal antibody targeting specifically IL-23, has been promising in clinical trials for CD, concerning patients with primary and secondary non-response to TNF- α antagonists. However, in June 2023, AstraZeneca announced that its development was discontinued due to commercial considerations rather than safety concerns [68]. Despite this suspension of research, the drug’s demonstrated efficacy and favorable safety profile suggest the potential for future studies’ resumption and the development of new therapeutic strategies.

In phase 2a trials, brazikumab significantly improved therapeutic response rates at week 8 in comparison to placebo (49.2% vs. 26.7%, *p* = 0.01) [21]. Research has shown that brazikumab treatment caused remarkably greater reductions in fecal calprotectin and CRP concentrations compared with placebo, confirming its efficacy in CD therapy [68]. The treatment was associated with an acceptable safety profile for over 100 weeks of therapy in patients with moderately to severely active CD who did not respond adequately or demonstrated intolerance to one or more TNF-α inhibitors [82].

A possible advantage of using brazikumab in CD treatment is the potential personalization of the therapy, based on baseline levels of IL-22 and CRP. A study indicates that the higher efficacy of brazikumab is likely to occur in patients with elevated baseline serum IL-22 levels or CRP. A personalized approach could use these data to predict patients’ responses to the therapy and optimize patient selection [83].

Future studies could explore brazikumab in combination with other agents, such as golimumab (a subcutaneous TNF-α antagonist), which is already under investigation with guselkumab [68], a strategy which might increase the efficacy of CD treatment by targeting multiple inflammatory pathways while maintaining safety.

Brazikumab could also be a potentially beneficial option for treating CD extra-intestinal manifestations (EIMs), such as psoriasis, but further research is needed to confirm these suggestions.

### 5.2. Tildrakizumab

Tildrakizumab is a humanized anti-IL-23p19 monoclonal antibody authorized for treating moderately to severely active plaque psoriasis. It has shown great efficacy in therapy for adult psoriatic patients, with an acceptable safety profile [84]. There is no available research regarding its usage in CD or UC treatment, although considering tildrakizumab’s similar target—IL-23p19—to other selective Il-23 inhibitors utilized in CD, the results of such studies could be favorable [85].

### 5.3. Oral Peptides Targeting Il-23R

The discovery of oral peptides like JNJ-77242113, which targets IL-23R, represents a promising complementary CD treatment strategy. JNJ-77242113 has shown favorable results in previous studies involving healthy volunteers and psoriasis patients and in a rat model of trinitrobenzene sulfonic acid-induced colitis. In a recently conducted phase 2 study of patients with moderately to severely active psoriasis, JNJ-77242113 achieved its primary clinical effectiveness objective and demonstrated a favorable safety profile. Therefore, oral alternatives like JNJ-77242113 could provide new, more convenient treatment options for patients with autoinflammatory diseases caused by dysregulated IL-23 signaling [86].

## 6. Conclusions

Crohn’s disease is a complex inflammatory condition, with a pathogenesis closely linked to proinflammatory cytokines, especially IL-23. Many studies have indicated the importance of this particle in CD genesis and maintenance, which has resulted in the creation of novel treatment strategies aimed at IL-23 and its associated receptors. The use of selective anti-IL-23 therapy results in significantly higher response rates in CD patients who have not responded efficiently to previous TNF-α antagonist treatment, which may be promising for the future management of these patients.

Risankizumab and guselkumab have both demonstrated higher remission rates and greater mucosal healing than ustekinumab. Risankizumab has shown compelling results in clinical trials, achieving remission rates of up to 45% during induction (ADVANCE trial) and 55% during maintenance therapy (FORTIFY trial). This therapy also demonstrated notable mucosal healing rates of 47% in long-term follow-up. Similarly, guselkumab has achieved remission in 40–50% of patients during induction, and 55–60% of patients during maintenance therapy, with endoscopic healing observed in 45%. Mirikizumab has proven effective in both induction and maintenance therapy, with a 48% endoscopic response and 54% clinical remission at 52 weeks, as shown in the VIVID-1 trial.

The described IL-23 inhibitors have demonstrated positive safety profiles with minimal rates of severe adverse effects both in the induction and maintenance phases. Commonly observed adverse effects included headache and nasopharyngitis. Risankizumab and guselkumab had slightly milder incidences of adverse events than mirikizumab.

In summary, the described IL-23 inhibitors have proven to be an effective and generally safe treatment for CD, with a potential to target this condition more specifically and successfully, especially in patients with refractory Crohn’s disease or those unresponsive to other biologics. They have demonstrated superior or comparable efficacy in achieving mucosal healing and endoscopic remission, evaluated against standard treatments. Additionally, the use of IL-23 inhibitors correlates with a reduced incidence of infections and malignancies in comparison to anti-TNF-α therapy, with significantly reduced risks of certain pathologies, such as encephalitis.

While further research is needed, particularly about the real-world effectiveness and long-term benefits and risks associated with using selective IL-23 inhibitors, available data make them an interesting new line of treatment, opening novel possibilities of finding more efficient CD management strategies.

### Limitations

This review has several limitations:

1. Database Scope—although multiple databases (PubMed, Embase, Cochrane Library, Web of Science) were used, relevant studies from other sources may have been missing.
2. Publication Bias—the focus on peer-reviewed articles may exclude unpublished or negative-result studies, potentially skewing findings.
3. Study Variability—differences in design, population, and outcome measures limit direct comparisons and generalizability.
4. Short-Term Data—most studies assess mid-term efficacy and safety, with limited long-term insights.
5. Confounding Factors—variations in medication use, disease severity, and genetic predispositions may impact results.
6. Real-World Evidence Gap—clinical trials dominate the data; real-world effectiveness needs further study.

Despite these limitations, IL-23 inhibitors show promise, warranting further long-term and real-world research.

## Figures and Tables

**Table 1:** Comparison of risankizumab treatment results across clinical studies.

Feature	D’Haens 2022 (ADVANCE Trial) [40]	D’Haens 2022 (MOTIVATE Trial) [40]	Fumery 2022 (GETAID) [42]	Peyrin-Biroulet 2024 (SEQUENCE Trial) [44]
Design of study	randomized, double-masked, placebo-controlled, phase 3 induction studies		retrospective cohort study	phase 3b, multicenter, open-label, randomized, controlled trial with blinded assessment of endpoints
CD activity	moderate-to-severe		moderate-to-severe	moderate-to-severe
Number of patients	850	569	100	230
Biologics failure history	58% intolerant of ≥1 biologic	47% intolerant of one biologic, 53% of more than one	100%	100%
Mean duration of CD	8.8 years	11.7 years	14.8 years	7.3
Dose of risankizumab	600 mg or 1200 mg		600 mg	600 mg
Clinical remission	42–45% ^a^ at week 12 (*p* < 0.0001)	40–42% ^a^ at week 12 (*p* < 0.0001)	58% (45.8% steroid-free) ^b^ at week 12	58.6% ^a^ at week 24
SF/AP remission	41–43% ^c^ at week 12 (*p* < 0.0001)	35–40% ^c^ at week 12 (*p* < 0.0001)	50% (39.5% steroid-free) ^c^ at week 12	no data
Endoscopic response	32–40% ^d^ at week 12 (*p* < 0.0001)	29–34% ^d^ at week 12 (*p* < 0.0001)	48% ^d^ at week 12	45.2% ^d^ at week 24 (*p* < 0.001)
Mean CRP change at week 12 (mg/L)	(−7.5)–(−10) (*p* < 0.0001)	(−10)–(−11) (*p* < 0.0001)	−3.1	no data
Mean FCP change at week 12 (mg/kg)	−1000 (*p* < 0.0001)	(−600)–(−1200) (*p* < 0.0001)	−1370	no data
Adverse events	51–56%	48–59%	20%	10.3%

^a^ CDAI < 150 points, ^b^ HB score < 5, ^c^ SF ≤ 3 and AP ≤ 1, ^d^ Decrease in SES-CD > 50% from baseline, Abbreviations: CD—Crohn’s disease, SF/AP—stool frequency and abdominal pain score, SES-CD—Simple Endoscopic Score for Crohn’s Disease, CRP—C-reactive protein, FCP—fecal calprotectin.

**Table 2:** The most common adverse effects observed with risankizumab treatment in patients with Crohn’s disease [40,42,46].

Adverse Effect	Frequency Range (%)	Description and Notes
Crohn’s disease worsening	11%	Aggravation of symptoms such as abdominal pain and diarrhoea
Nasopharyngitis	6%	Inflammation of the nose and throat
Arthralgia	5–15%	Joint pain or discomfort
Headache	3–6%	A feeling of pain or pressure in the head
Viral upper respiratory tract infection	6%	Common cold symptoms including cough
Nausea	1–5%	A sensation of discomfort in the stomach with an urge to vomit
Abdominal pain	1–5%	Pain or cramping in the stomach or abdominal region
Diarrhoea	1–3%	Frequent, loose or watery stools
Anaemia	2–5%	Reduced haemoglobin
Injection site reaction	1–2%	Redness, swelling, or irritation at the location of drug injection
Otitis	1%	Inflammation of the ear
Skin rash	1%	Red, irritated, or inflamed skin
Bronchitis	1%	Inflammation of the bronchial tubes, often causing coughing and difficulty breathing
Alopecia	1%	Loss of hair
Mastodynia	1%	Breast pain or tenderness
Hypertension flare-up	1%	Increase in blood pressure

**Table 3:** Comparison of guselkumab treatment results across clinical studies.

Feature	Sandborn 2021 (GALAXI 1 Trial) [55]	Panés 2025 (GALAXI 2 and 3 Trial) [62]
Design of study	Randomized, double-blind, placebo-controlled, phase 2 induction study	Phase 3, multicenter, randomized, double-masked study
CD activity	moderate-to-severe	
Number of patients	250	1021 (508 in G2, 513 in G3)
Biologics failure history	65% intolerant of ≥1 biologic	52.8% BIO-IR, 41.9% BIO-naive
Mean duration of CD	9.2 years	8.5 years
Dose of guselkumab	200 mg, 600 mg, 1200 mg	200 mg IV q4w (×3) → 100 mg SC q8w or 200 mg SC q4w
Clinical remission	40–50% at week 12 (*p* < 0.0001)	
Endoscopic response	35–45% at week 12 (*p* < 0.0001)	47–52% (*p* < 0.0001)
Corticosteroid-free endoscopic response at Week 48	41.3–42.5%	
Corticosteroid-free clinical remission at Week 48	52.3–54.7%	
Adverse events	50–57%	46–55%

Abbreviations: CD—Crohn’s disease, BIO-IR—Biologic-Inadequate Responder, BIO-naive—Biologic-Naive, q4w—every 4 weeks, q8w—every 8 weeks, SC—subcutaneous.

**Table 4:** Adverse events reported with guselkumab treatment in patients with Crohn’s disease [54,55].

Adverse Effect	Frequency Range (%)	Description and Notes
Nasopharyngitis	11%	Nasal congestion and discharge, moist and productive cough, sneezing, and sore throat
Upper respiratory infections	6%	May include symptoms like cough, nasal congestion, and sore throat
Headache	5–15%	Mild to moderate intensity headaches reported
Herpes zoster	58%	Reactivation of a dormant VZV quiescent
Encephalitis	18%	Brain inflammation
Injection Site Reactions	5–15%	Includes redness, itching, pain, or swelling at the injection site
Fatigue	5–10%	General tiredness or low energy, which may vary in intensity
Nausea	3–7%	Nausea or upset stomach reported in some patients.
Serious Infections	<1%	Rare, but includes potential for more serious infections (e.g., tuberculosis)
Allergic Reactions	Rare	Includes skin rash or hypersensitivity reactions; rare but possible

**Table 5:** Comparison of mirikizumab treatment results across clinical studies.

Feature	Sands 2022 (SERENITY Trial) [75]	Ferrant 2024 (VIVID-1 Trial) [76]
Design of study	Phase 2 multicenter, randomized, parallel-arm, double-blind, placebo-controlled trial	Phase 3, randomized multicentre, double-blind, placebo-controlled and active-controlled, treat-through study
CD activity	moderate-to-severe	
Number of patients	191	1150
Biologics failure history	56.3% to at least one biologic	30.2% intolerant of one biologic, 18.3% of more than one
Mean duration of CD	10.2 years	7.4 years
Dose of mirikizumab	200 mg, 600 mg, and 100 mg iv,	900 mg iv.
Clinical remission ^a^	26.6% ^e^ (*p* < 0.05) to 40.6% ^d^ (*p* < 0.001) at week 12	37.7% at week 12 (*p* < 0.0001)
Endoscopic ^b^ remission	15.6% ^d^ (*p* < 0.05) to 20.3% ^e^ (*p* < 0.01) at week 12	10.9% at week 12 (*p* = 0.0034)
Endoscopic ^c^ response	37.5% ^d^ (*p* < 0.01) to 43.8% ^e^ (*p* < 0.001) at week 12	32.5% at week 12 (*p* < 0.0001)
HsCRP change at week 12 from baseline	−29.9% ^d^ to −48.6% ^e^ at week (*p* < 0.001)	No data
FCP change at week 12 from baseline	−62.1% ^d^ (*p* < 0.05%) to −76.2% ^e^ (*p* < 0.001)	No data
Adverse events	70.3%	78.6%

^a^ CDAI < 150 points, ^b^ total SES-CD score of ≤4 and a minimum 2-point reduction from baseline with no subscore > 1, ^c^ at least 50% reduction in SES-CD total score from baseline, ^d^ 600 mg iv group, ^e^ 1000 mg iv group. Abbreviations: CD—Crohn’s disease, hsCRP—high specific C-reactive protein, FCP—fecal calprotectin, CDAI—Crohn’s Disease Activity Index, SES-CD—Simple Endoscopic Score for Crohn’s Disease, iv—intravenous.

**Table 6:** Adverse effects observed with mirikizumab treatment in patients with Crohn’s disease [75,76].

Adverse Effect	Frequency Range (%)	Description
Headache	5–15%	A feeling of pain or pressure in the head
COVID-19	16.5%	Infection caused by SARS-CoV-2 virus.
Nasopharyngitis	5–13%	Inflammation of the nose and throat
Arthralgia	3–13%	Joint pain or discomfort
Injection Site Pain	3–9%	Pain at the injection site
Anemia	3–8%	Reduced haemoglobin
Upper respiratory infections	5–7%	Common cold symptoms including cough
Abdominal pain	5–7%	Pain or cramping in the stomach or abdominal region
Weight increase	3–7%	Rise of body mass
Nausea	3–6%	A sensation of discomfort in the stomach with an urge to vomit
Crohn’s disease worsening	3%	Aggravation of symptoms such as abdominal pain and diarrhea
Serious adverse invents	4%	Serious adverse effects include chest pain, colon stenosis, and colon perforation)

## Data Availability

No new data were created or analyzed in this study. Data sharing is not applicable to this article.

## Abbreviations

AD	adjusted difference
AIEC	adherent-invasive *Escherichia coli*
AP	abdominal pain score
APCs	antigen presenting cells
CDAI	Crohn’s Disease Activity Index
CD	Crohn’s disease
CI	confidence interval
CRP	C-reactive protein
DCs	dendritic cells
FCP	fecal calprotectin
GCS	glucocorticoids
GM-CSF	granulocyte-macrophage colony-stimulating factor
GWAS	genome-wide association study
HB	Harvey–Bradshaw index
IBD	inflammatory bowel disease
IgG1	immunoglobulin G1
IL-23	interleukin-23
IL-23R	interleukin-23 receptor
IRR	incidence rate ratio
JAK	Janus kinase
MAP	*Mycobacterium avium* subspecies paratuberculosis
MMPs	matrix metalloproteinases
OR	odds ratio
PCR	polymerase chain reaction
SAEs	serious adverse events
SES-CD	Simple Endoscopic Score for Crohn’s Disease
SF	stool frequency
SNPs	single nucleotide polymorphisms
TDM	therapeutic drug monitoring
TEAEs	treatment-emergent adverse effects
Th17	T helper 17
TNF	tumor necrosis factor
TNF-α	tumor necrosis factor-alpha
TYK2	tyrosine kinase 2
